# Rethinking Sustainability in Plant-Based Proteins: A Systems Perspective from Crop to Consumer

**DOI:** 10.3390/foods15142433

**Published:** 2026-07-09

**Authors:** William R. Aimutis, Carlos Iglesias, Marvin Moncada, Andrew Neilson, Haotian Zheng, Rohan A. Shirwaiker

**Affiliations:** 1 Bezos Center for Sustainable Protein, North Carolina State University, Fitts-Woolard Hall, 915 Partners Way, Raleigh, NC 27695, USA; 2 Department of Crop and Soil Sciences, Plant Breeding Consortium, North Carolina State University, Kilgore Hall, 2721 Founders Drive, Raleigh, NC 27607, USA; cainglesi@ncsu.edu; 3 Department of Food, Bioprocessing, and Nutrition Science, North Carolina State University, Schaub Food Science Building, 400 Dan Allen Dr., Raleigh, NC 27607, USA; mlmoncad@ncsu.edu (M.M.); aneilso@ncsu.edu (A.N.); haotian.zheng@ncsu.edu (H.Z.); 4 Edward P. Fitts Department of Industrial Systems Engineering, North Carolina State University, Fitts-Woolard Hall, 915 Partners Way, Raleigh, NC 27695, USA

**Keywords:** plant-based, protein, sustainability, functionality, food safety, supply chain, nutrition, climate

## Abstract

Plant-based protein (PBP) foods are increasingly promoted as sustainable alternatives to animal-derived proteins, yet their environmental and nutritional performance depends on complex interactions among crop selection, agricultural practices, processing technologies, formulation strategies, food safety controls, and climate variability. This review synthesizes evidence across the entire plant-based protein value chain, examining how crop physiology, protein extraction and structuring, ingredient functionality, safety risks, and nutritional outcomes are shaped by both technological choices and environmental changes. Particular attention is given to climate-induced alterations in protein concentration, amino acid composition, mineral bioavailability, and contaminant risk, and how these upstream changes propagate through processing and food formulation to affect consumer health outcomes. We argue that sustainability is an emergent system-level outcome determined by interactions among biological, technological, nutritional, and socio-economic factors rather than an intrinsic property of any given crop or protein source. The review concludes by identifying research, processing, and policy priorities needed to enable scalable, climate-resilient, safe, and nutritionally robust plant-based protein systems.

## 1. Introduction

Food production systems are fundamental to human survival, health, and economic development, yet they are also major drivers of environmental degradation. The global food system, from agricultural inputs through processing, distribution, consumption, and waste, contributes substantially to greenhouse gas emissions, land conversion, biodiversity loss, and freshwater depletion [1]. Among food categories, animal-derived proteins are especially resource-intensive due to low feed-conversion efficiency, high land and water demands, and methane emissions from ruminant livestock.

In light of these challenges, plant-based foods and plant-based proteins (PBP), in particular, have been widely encouraged as more sustainable dietary alternatives [2]. Life-cycle assessment (LCA) studies consistently demonstrate lower greenhouse gas emissions (GHG*e*) and land requirements for plant-based foods relative to comparable animal products [3,4,5,6]. These findings continue to inform dietary guidelines, national sustainability strategies, and private-sector innovation in alternative proteins. Over the last decade, global sales of plant-based meat, dairy, and protein ingredients have grown rapidly, accompanied by significant investment in processing and product development.

However, the PBP markets have slowed in recent years in several regions. Consumer concerns have emerged regarding cost, sensory quality, cultural relevance, nutritional adequacy, and the perceived “ultra-processed” nature of many plant-based alternatives [7]. These challenges highlight a key limitation of prevailing sustainability narratives, which often emphasize environmental performance without sufficient consideration of nutrition, processing complexity, food safety, and resilience to climate variability.

**Figure 1:**
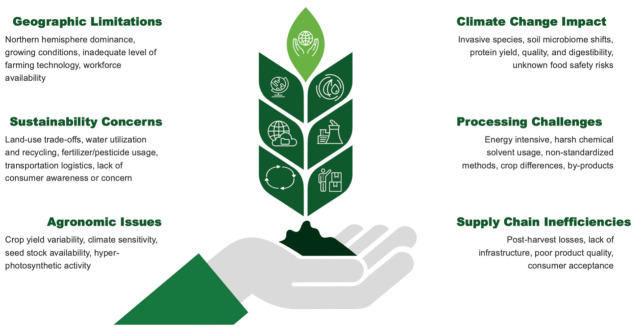
System components impacting the environmental sustainability of plant-based protein-rich food formulations.

Sustainability in food systems cannot be reduced to a single metric or input (Figure 1). Rather, it emerges from interactions across the value chain, with crop genetics and agronomic practices shaping raw material composition, processing technologies influencing energy use, ingredient functionality, and safety, and formulation choices affecting nutrient delivery and sensory quality. Treating sustainability as an intrinsic property of plant-derived proteins obscures these interactions and risks over-simplifying the transition away from animal-based foods.

Climate change contributes to the complexity across the value chain. Elevated atmospheric CO_2_ concentrations, rising temperatures, altered precipitation regimes, and increasing frequency of extreme weather events are already altering crop physiology. These changes affect protein concentration, amino acid composition, and mineral density, phenomena collectively described as carbon dilution and stress-induced compositional shifts [8,9,10]. While the agronomic impacts of climate change are increasingly well documented, the downstream consequences for protein extraction, ingredient functionality, food safety, and nutritional quality remain relatively underexplored.

This paper examines the sustainable production of PBP foods as an integrated system, from crop selection through processing, functionality, safety, and nutrition. We emphasize how climate variability reshapes each stage of the PBP value chain, and why sustainability must be understood as a dynamic, emergent property rather than a fixed attribute of plant-derived proteins.

## 2. Protein-Rich Crop Systems in the Alternative Protein Value Chain

### 2.1. Key Crops for Plant-Based Proteins

The alternative protein sector currently relies on a relatively narrow suite of protein-rich plant crops, predominantly legumes and oilseeds [11,12]. Key nutrient characteristics of protein-rich crops are summarized (Table 1).

**Figure 2:**
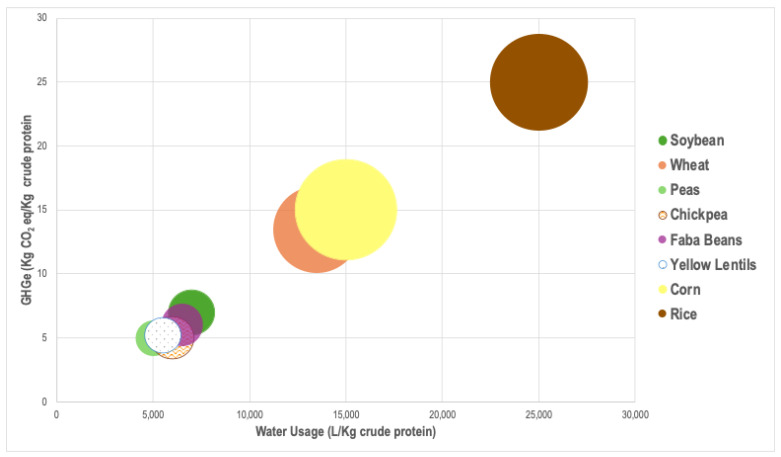
Environmental impact of crude protein synthesis by crops commonly cultivated for commercially available, sustained protein concentrates and isolates. Pulses and other nitrogen-fixing crops are less environmentally harsh than cereals that require more water and are negatively correlated with GHG*e*. Generally, GHG*e* < 2 Kg CO_2_ *eq*/Kg crude protein and water usage of <5000 L/Kg crude protein are considered environmentally most favorable, while GHG*e* and water usage of 2–5 Kg CO_2_ *eq* and 5000–15,000 L/Kg, respectively, are considered environmentally adequate [38]. For comparison, beef cattle produce approximately 120–205 Kg CO_2_ *eq*/Kg crude protein while only using approximately 15,500 L of water [39,40].

Plant growth and protein accumulation are governed by a tightly linked set of agronomic and environmental factors, with nitrogen availability playing a central role in driving amino acid synthesis and overall protein content (Table 2). Adequate sunlight (photosynthetically active radiation) and optimal temperatures regulate photosynthesis and carbon–nitrogen balance, while water availability influences nutrient transport, metabolic activity, and stress responses that can either enhance or dilute protein concentration [33]. Soil health—including structure, organic matter, and microbial activity—supports nutrient cycling and root development, enabling efficient uptake of nitrogen, sulfur, and micronutrients essential for protein formation [34]. Finally, agronomic practices such as cultivar selection, planting density, and timing of fertilization interact with these environmental conditions to determine the efficiency of protein filling during critical growth stages like seed development [35,36,37]. Reluctantly, many of these same factors contribute to the negativity associated with environmental impact. For example, three major crops globally cultivated [corn (*Zea mays*), rice (*Oryza sativa*), and wheat (*Triticum aestivum* or *Triticum durum*)] have a very high-water demand and emit more GHG*e* than other food production crops (Figure 2). Fortunately, the cultivation of protein-rich crops is not as negatively impactful to the environment as animal agriculture.

**Table 2:** Environmental Elements Needed During Plant Cultivation to Optimize Protein Synthesis.

Crop	Preferred Soil and pH	Global Harvest (MMT)	Typical Total Crude Protein (%)	Acreage Cultivated to Yield 1 MT of Crude Protein (Acres)	Soil Nutrient Required/MT TCP (Kg)	Water Demand (MM Liters/MT TCP)	Range and Optimum Daytime Growing Temperature (°C)
Soybean	Well-drained loam or silt loam, pH 6.0–7.5	~422	~35–40	1.2–1.3	N: 80 P: 25 K: 53	3.5–6.0	Range: 20–30 Optimum: 29–30
Wheat	Well-drained loam or clay loam, pH 6.0–7.5	818–837.8	12–14	2.5–4.0	N: 100–150 P: 20–30 K: 25–40	10.0–15.0	Range: 15–24 Optimum: 21–24
Yellow Pea	Sandy loam or texture loam, pH 5.5–7.5	12.6 (includes green peas)	20–25	1.6–2.2	N: 15–40 P: 2–4 K: 9	7.9–9.9	Range: 13–21 Optimum: 15–24
Rice	Heavy clay or silt loam with high water retention (preferably flooded), pH 5.0–7.5	770	7–8	4.5–5.5	N: 130–170 P: 30–40 K: 130–170	175.0–350.0	Range: 15–38 Optimum: 21–31
Chickpea	Well-drained sandy loam to silt loam, pH 6.0–7.5	18.1	18–25	4–6	N: 10–20 P: 50–60 K: 20–40	35.0–40.0	Range: 10–29 Optimum: 21–27
Faba Bean	Heavy silt or clay loams, pH 6.0–7.0	5.67	25–30	1.1–2.5	N: 4.5–9.1 P: 22.7–68.2 K: 22.7–54.5	6.0–8.0	Range: 4.5–27.0 Optimum: 15–21
Yellow Lentil	Well-drained, deep, sandy loam; pH 6.0–7.0	6.6–6.7	24.5–27.5	3.3–4.5	N: 0–17 P: 112–168 K: 56–112	4.9–5.0	Range: 4–29 Optimum: 15–24
Corn	Deep, well-drained sandy loam or loamy soil, pH 6.0–6.8	1,233	16.5	1.2–1.5	N: 150–200 P: 60–80 K: 70–90	9.0–12.0	Range: 10–35 Optimum: 24–32

References: Soybean: [41,42,43,44,45,46,47,48,49]; Wheat: [43,50,51,52,53,54,55]; Yellow peas: [43,56,57,58,59,60]; Rice: [61,62,63,64,65]; Chickpea: [43,66,67,68,69]; Faba bean: [43,56,70,71,72,73,74,75,76]; Yellow lentil: [43,56,77,78,79]; Corn: [43,80,81,82,83].

Soybean (*Glycine max*) remains the most widely used crop for PBP ingredients due to its high protein concentration, relatively balanced essential amino acid profile, and extensive global production and processing infrastructure [84]. Soybeans are compatible with existing oilseed crushing and fractionation systems, facilitating large-scale production of protein concentrates and isolates.

Despite these advantages, inputs for soybean and other high-protein crop production have raised sustainability concerns. Large-scale monocultures, particularly in Brazil, have contributed to soil degradation, nutrient runoff, and, in some regions, deforestation and habitat loss [85]. Brazilian soybean cultivation has displaced over 1 million hectares of rainforest. The consequences of monocultured crops are reductions in native habitat heterogeneity, including native plants, insects, birds, mammals, and soil microbiome. The widespread adoption of genetically modified soybean varieties has increased yields and simplified weed management, but has also contributed to biodiversity loss and herbicide resistance [86]. In addition, soy is classified as a major food allergen, and uncertainties remain regarding the classification of major and minor soy allergens, variability in allergen expression across cultivars, and the effects of processing on allergenicity [87].

Other legumes, including peas (*Pisum sativum*), lentils (*Lens culinaris*), chickpeas (*Cicer arietinum*), and faba beans (*Vicia faba*), have gained prominence due to their agronomic and sustainability advantages [6,88,89,90]. These crops fix atmospheric nitrogen, integrate well into crop rotations, and generally require lower inputs of synthetic fertilizer [91]. Peas and faba beans perform well in temperate climates, whereas lentils and chickpeas are well suited to semi-arid regions and dryland farming systems.

However, except for soybean, most of these legumes contain protein concentrations below 30% on a dry matter basis (Table 1). As a result, protein extraction generates large volumes of starch-rich byproducts, increasing processing demands and necessitating effective co-product valorization to maintain economic and environmental sustainability. These trade-offs illustrate that crop diversification alone does not guarantee improved sustainability outcomes; downstream processing burdens must also be considered.

### 2.2. Environmental Performance and Agronomic Considerations

Environmental impacts of protein crop production vary widely across regions and management systems (Table 3). Soil characteristics, climate, cultivar genetics, irrigation intensity, fertilizer regimes, and pest pressure all influence GHG*e*, nutrient losses, and water footprints [89]. Crop rotation, intercropping, and reduced-tillage practices can enhance soil structure, reduce erosion, and lower dependence on synthetic inputs [6,85]. These approaches are particularly effective for legumes, which contribute biologically fixed nitrogen to subsequent crops.

Regenerative agricultural practices, including cover cropping, residue retention, and reduced disturbance, have been implemented in some soybean and pulse production systems to mitigate erosion and enhance soil carbon sequestration [117]. However, outcomes remain highly context-dependent and are influenced by market incentives, infrastructure, and policy support. Controlled-environment agriculture and bioreactor-based systems have been proposed for certain protein biomass sources, including algae and fungi that leverage agricultural side streams as feedstocks, but their energy demands and scalability introduce unresolved sustainability trade-offs [6].

## 3. Protein Concentration, Isolation, and Structuring Technologies

Plant-based proteins are embedded in complex cellular matrices that contain fiber, starch, lipids, phenolic compounds, pigments, and numerous other components, making them comparatively more challenging than animal-sourced proteins for extraction and concentration. The technologies discussed below enable increased protein density, selectivity of functionality, and reduction of anti-nutritional factors and off-flavors to provide protein concentrates and isolates with sensorial and nutritional properties comparable to animal products. Ultimately, the technology selected for protein purification impacts sustainability and bilaterally influences the ease and challenge of food formulation and product quality.

### 3.1. Protein Concentration and Isolation

Protein isolation has emerged as a central technological platform enabling the transition toward plant-based protein systems. Protein concentration and isolation processes transform heterogeneous crop materials into functional protein ingredients that serve as the building blocks for nutritious, organoleptically acceptable food products. Two primary routes are employed: dry fractionation and wet extraction.

**Figure 3:**
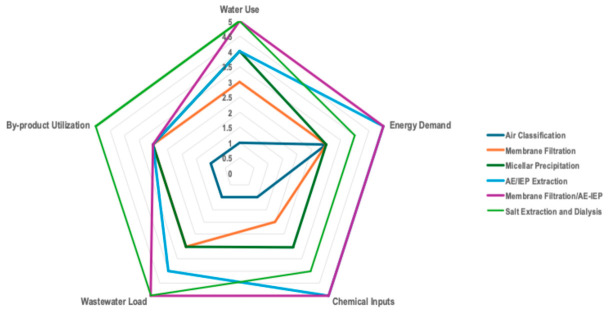
Sustainability comparison of protein extraction and concentration methods. Values: 1: excellent; 2: good; 3: adequate; 4: poor; and 5: very poor. As observed from the figure, air classification is the most environmentally favorable method, and salt extraction and dialysis are the least. Air classification yields lower protein concentrations than other methods [119,120].

Dry fractionation methods, including milling followed by air classification or electrostatic separation, exploit physical differences between protein-rich and starch-rich particles [118]. These processes require minimal water and energy, generate no liquid effluents, and preserve some native functional properties (Figure 3). However, dry-fractionated protein concentrates typically achieve only moderate protein purities (approximately 50–65%) and retain significant amounts of starch, fiber, and lipids that can limit protein functionality and introduce variability in flavor, color, and processing performance. Consequently, these protein products are often used in bakery, snack, and hybrid formulations rather than for highly structured meat alternative applications.

Wet extraction methods—most commonly alkaline solubilization followed by isoelectric precipitation—enable production of higher-purity protein concentrates and isolates, often exceeding 80% protein. These methods improve solubility and standardize functional performance but are water- and energy-intensive and frequently induce protein denaturation and aggregation [121]. Alternative wet processes, such as salt extraction or membrane filtration, offer milder conditions but present challenges related to fouling, yield, and scalability (Figure 3). The higher protein purity achieved through wet extraction is particularly important in applications requiring functional properties of gelation, emulsification, water-holding capacity, and fibrous structure formation. 

The transition from protein concentrates (60–80% protein) and isolates (>80% protein) represents more than an increase in protein content. Removal of non-protein components improves protein functionality by eliminating protein interaction with fibers, starch, or lipids. Fewer non-protein components enable more protein–protein interaction to improve manipulation of functional properties, resulting in products with improved texture, appearance, and sensory consistency. Concentrates often retain co-components that support texture and emulsification, while isolates are favored for their consistency, neutral flavor, and versatility across applications such as meat and dairy analogs.

The success of downstream structuring technologies is highly dependent on the composition and purity of the protein ingredient. Protein ingredients will unfold, aggregate, and reorganize their structure into continuous networks when thermally or mechanically processed. Consequently, protein isolation is not merely a purification step but a prerequisite for many emerging texturization technologies being employed to recreate highly ordered structures found in animal tissues.

### 3.2. Protein Structuring and Texturization

Texturization is essential for producing plant-based meat analogs with fibrous, anisotropic structures [122]. Extrusion remains the most established structuring technology, using combinations of heat, moisture, pressure, and shear to align proteins into fibrous networks. Low-moisture extrusion produces texturized vegetable protein (TVP), whereas high-moisture extrusion yields structures resembling whole-cut meats.

Shear-cell technologies represent an emerging alternative, using controlled laminar shear and moderate thermal input to induce protein alignment with lower specific energy consumption [122]. Although less mature at an industrial scale, shear-based methods offer potential advantages in energy efficiency and structural control [123].

Biological protein production routes, such as fungal fermentation, differ fundamentally from post-extraction structuring [6]. Mycoprotein and other biomass-based proteins acquire fibrous texture during growth rather than through applied mechanical forces and therefore follow distinct value chains with different sustainability considerations.

## 4. Ingredient Functionality and the Colloidal State of Plant Proteins

Functional performance of plant-based protein ingredients is a primary determinant of product quality, consumer acceptance, processing feasibility, and overall sustainability of plant-based foods. Unlike animal proteins, which evolved to function within aqueous physiological environments (e.g., milk, blood, muscle tissue) [124], most plant proteins are storage molecules accumulated in seeds to support germination [125]. Their biological role is therefore to remain compact, stable, and largely insoluble under dry conditions, rather than to participate in interfacial stabilization, gelation, or emulsification. This fundamental difference underpins many of the functional challenges encountered when plant proteins are repurposed as food ingredients.

From the perspective of food structure design, plant protein functionality is best understood using concepts from colloid and interface sciences [126,127,128]. Functional properties emerge not from protein chemistry alone, but from multiscale interactions spanning molecular conformation, mesoscale aggregation, and macroscopic structuring [129]. Many performance limitations observed in PBP foods—including sedimentation in beverages, chalky mouthfeel, coarse gel texture, or unstable emulsions—are manifestations of unfavorable colloidal states rather than intrinsic shortcomings of plant proteins themselves [130,131].

### 4.1. Multiscale Structure–Function Relationships

Plant protein functionality manifests across multiple hierarchical length scales, each contributing distinct constraints and opportunities for food formulation [129,132].

At the molecular scale, amino acid composition, charge distribution, hydrophobicity, and conformational flexibility determine intrinsic properties such as solubility, isoelectric point, enzymatic accessibility, and bitterness perception [129]. Storage proteins such as globulins, prolamins, and glutelin typically exhibit compact tertiary and quaternary structures stabilized by hydrophobic interactions and disulfide bonds. These stabilizing forces reduce hydration and limit unfolding at food-relevant conditions, thereby restricting interfacial activity and network formation when compared with more flexible animal proteins [132].

At the mesoscopic length scale, plant proteins self-assemble into oligomers, aggregates, or particulate structures (e.g., fibrils) ranging from tens of nanometers to one micrometer. These mesoscale assemblies act as colloidal building blocks that govern viscosity, interfacial adsorption kinetics, rheological behavior under shear, and phase stability [131]. In many plant protein dispersions, soluble monomers coexist with insoluble aggregates in a dynamic equilibrium that is sensitive to pH, ionic strength, temperature, and processing history [130,133,134].

The mesoscale is particularly critical for plant-based foods because it links molecular structure to macroscopic performance [132]. For example, large, aggregated particles sediment under gravity in beverages, while smaller, deformable aggregates may behave as effective interfacial stabilizers [133]. Many aforementioned quality defects observed in PBP foods—such as phase separation in emulsions, excessive viscosity, or gritty mouthfeel—originate from poorly controlled mesoscale particle populations rather than from chemical limitations of the protein itself [135].

At the macroscopic scale, protein assemblies interact with lipids, carbohydrates, and water to form gels, emulsions, foams, and fibrous networks [136,137]. Mechanical properties such as elasticity, cohesiveness, fracture behavior, and lubrication are governed by how mesoscale particles percolate, deform, and crosslink within these bulk structures [138,139,140,141]. Importantly, optimal macroscopic performance does not necessarily require complete protein solubility. In structured foods such as meat analogs, partial insolubility and controlled aggregation are often essential for developing fibrous, meat-like textures [122].

### 4.2. Influence of Protein Extraction on Colloidal State

Protein extraction is a defining step in shaping the colloidal state of plant protein ingredients and, consequently, their functional behavior [132]. The dominant industrial method—alkaline extraction followed by isoelectric precipitation (AE-IP)—involves solubilization of proteins at alkaline pH, followed by precipitation near their isoelectric point [142,143]. While effective for achieving high protein purity, this approach profoundly alters protein structure.

During alkaline solubilization, hydrogen bonds, electrostatic interactions, and hydrophobic packing that stabilize native conformations are disrupted [143]. Subsequent acidification to the isoelectric point promotes aggregation through reduced electrostatic repulsion, exposing hydrophobic residues and reactive sulfhydryl groups [144]. Aggregation is further intensified during washing, neutralization, and drying steps. As a result, many commercial protein isolates consist largely of modified and aggregated protein particles rather than proteins in their original colloidal states in the parental plant sources [132,143,145].

Although preservation of native structure is often assumed to improve functionality, empirical evidence suggests this assumption is overly simplistic. Mild extraction procedures may retain higher proportions of native protein but can yield ingredients with low solubility, limited interfacial adsorption, or weak gelation [132]. Conversely, controlled pre-denaturation can generate protein particles with improved surface activity, water-binding capacity, or network-forming ability, depending on the target application [146,147,148].

Dry fractionation methods provide a contrasting pathway. By avoiding extreme pH shifts and chemical solvents, dry fractionation preserves more native protein structure but typically yields mixed fractions containing starch granules, insoluble fibers, and residual lipids [149]. These co-components can be advantageous in some applications, contributing to viscosity, water retention, and texture. However, they can also introduce thermodynamic incompatibility between biopolymers, leading to phase separation, poor physical stability, or inconsistent textural performance [150,151]. Understanding the behavior of these mixed biopolymer systems is therefore essential for the effective application of dry-fractionated proteins.

### 4.3. Protein Aggregation: Liability or Opportunity?

Protein aggregation is frequently framed as a liability in PBP ingredients, particularly in applications where solubility and colloidal stability are prioritized, such as beverages or dairy alternatives. Indeed, excessive aggregation can reduce hydration efficiency, promote sedimentation, and generate undesirable sensory attributes [143]. However, aggregation is not inherently detrimental; its functional impact depends on aggregate size, morphology, reversibility, and surface chemistry [133,152,153,154,155].

Controlled aggregation induced by thermal, mechanical, or chemical treatments can be harnessed to improve functionality [156,157,158,159,160,161,162]. Protein aggregates with appropriate size and surface properties can function as Pickering or “Mickering” stabilizers, anchoring oil–water interfaces and enhancing emulsion stability [134,152,163]. Aggregated particles can act as gelation nuclei, lowering the critical protein concentration required for network formation [144]. In fibrous meat analogs, aggregated proteins contribute to anisotropic network formation and mechanical strength [164].

Problems arise when aggregation is uncontrolled. During spray drying, for example, protein particles with high surface hydrophobicity may undergo secondary aggregation, leading to poor rehydration and persistent insolubility [165]. Storage-induced aggregation can further degrade functionality over time [166]. Rehydration behavior—often insufficiently characterized in ingredient specifications—is therefore a critical determinant of industrial applicability.

Strategies to mitigate aggregation-related drawbacks include controlled pre-aggregation to define particle populations, inclusion of stabilizing excipients, or post-drying particle engineering [167,168,169]. These approaches illustrate that the goal is not the elimination of aggregation, but control over the aggregation pathway and final colloidal state.

### 4.4. Interfacial Behavior and Mixed Biopolymer Systems

In most plant-based foods, proteins do not function in isolation but interact with lipids, starch, dietary fiber, and low-molecular-weight solutes. These interactions determine interfacial structure, rheology, and stability [137,170]. Compared with animal proteins, many plant proteins exhibit slower adsorption kinetics at interfaces and form weaker interfacial films, necessitating higher concentrations or blending with other ingredients to achieve comparable performance [171].

Electrostatic interactions, steric hindrance from polysaccharides, and competitive adsorption at interfaces further complicate performance in mixed systems [172]. Incompatibility between protein-rich and carbohydrate-rich phases can drive phase separation unless formulation and processing are carefully optimized [173,174]. These phenomena underscore the importance of system-level formulation strategies rather than protein-centric optimization alone.

### 4.5. Lack of Standardization in Functional Metrics

A persistent methodological limitation in plant-based protein research is the absence of standardized protocols for functional assessment. Reported solubility values vary widely across studies due to inconsistent centrifugation forces, pH conditions, protein concentrations, and definitions of “soluble” protein [175]. Similar inconsistencies exist for emulsifying activity, foaming capacity, and gelation measurements.

This lack of standardization hampers cross-study comparison, obscures structure–function relationships, and slows rational ingredient development. For the field to advance, functional testing methods must be harmonized and aligned with application-relevant performance criteria rather than arbitrary analytical convenience [176]. Establishing such standards is essential for translating laboratory insights into scalable, industrially robust solutions [125,177,178].

## 5. Climate-Driven Impacts Across the Plant-Based Protein Value Chain

Climate change exerts multifaceted effects on PBP systems, influencing not only crop yields but also protein composition, ingredient functionality, food safety, and nutritional value. These impacts propagate across the entire value chain, from agricultural production through protein extraction, processing, and formulation, ultimately shaping the sustainability and performance of PBP foods. Importantly, many climate-driven effects are subtle, incremental, and interactive, making them difficult to detect using traditional yield-centric metrics yet highly consequential for downstream processing and nutrition.

### 5.1. Climate Effects on Protein Yield and Composition

Elevated atmospheric CO_2_ concentrations enhance photosynthetic carbon assimilation in many crops, increasing carbohydrate accumulation and biomass production. However, this stimulation of carbon fixation is frequently accompanied by a relative reduction in nitrogen assimilation, resulting in decreased protein and mineral concentrations, i.e., the carbon dilution effect [8,9,10]. This effect has been observed across major cereal, legume, and oilseed crops widely used in plant-based protein applications, including wheat, rice, barley, soybean, peas, and lentils [121,179,180,181,182].

While nitrogen-fixing legumes partially offset protein dilution through symbiotic nitrogen fixation, evidence increasingly suggests that protein concentration and quality may still decline under prolonged elevated CO_2_ exposure [183]. In several crops, elevated CO_2_ has been associated not only with reduced total protein concentration but also with shifts in amino acid composition, including reductions in lysine, methionine, and other essential amino acids (Table 1). Such changes degrade protein quality even when crude protein levels remain within historical ranges.

Temperature stress further modifies storage protein synthesis and composition [184]. Heat stress during flowering and seed filling disrupts transcriptional and translational regulation of storage proteins, promoting protein degradation pathways and altering the relative abundance of protein fractions [185,186]. In cereals, this manifests as altered glutenin-to-gliadin ratios, modifying viscoelastic properties relevant to food structuring [187,188]. In legumes and oilseeds, shifts among globulin and albumin fractions affect solubility, gelation behavior, and extraction efficiency [189,190,191,192].

Water stress compounds these effects by limiting nitrogen uptake and transport while accelerating senescence and stress metabolism [193,194]. Drought-stressed plants often accumulate free amino acids and stress proteins at the expense of storage protein synthesis, further reducing protein yield and altering fractionation profiles [195,196]. Importantly, combined stressors—such as simultaneous heat and drought—often produce non-additive effects that differ qualitatively from single-factor responses, highlighting limitations of climate studies that examine CO_2_, temperature, or water stress in isolation [197,198].

### 5.2. Implications for Protein Extraction and Ingredient Yield

Climate-induced variability in protein concentration and composition directly affects protein extraction efficiency and yield [199,200,201]. Reduced protein concentration increases the mass of raw material required to produce a given quantity of protein concentrate or isolate, increasing energy use, water consumption, and byproduct generation per unit of protein produced. Mindful selection of the extraction method could minimize the adverse environmental consequences of extra processing (Figure 3). This effect undermines assumptions of stable processing yields embedded in many sustainability and techno-economic analyses.

Altered protein fractionation further complicates extraction. Shifts in storage protein ratios influence solubility behavior under alkaline or salt extraction conditions and modify precipitation dynamics during isoelectric recovery [202,203,204,205,206]. Proteins synthesized under climate stress may exhibit altered isoelectric points, aggregation tendencies, and susceptibility to denaturation, requiring higher processing intensity to achieve comparable yields and purities [187,207,208].

**Figure 4:**
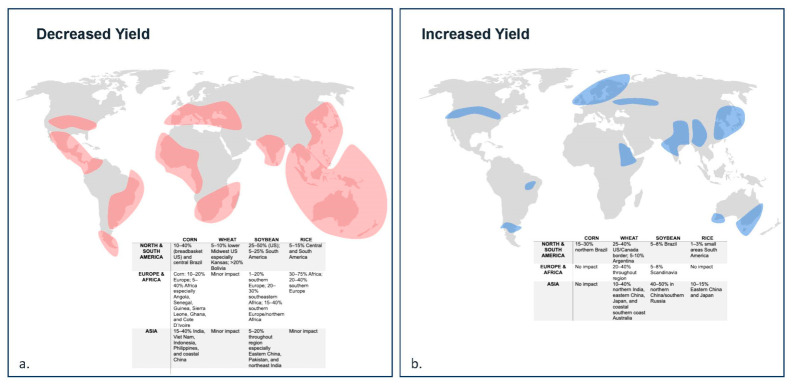
Projected impacts of climate change on protein crop productivity worldwide. **(a**) Regions experiencing decreased yields (red shading) due to temperature stress and altered precipitation patterns. **(b**) Regions showing increased yields (blue shading) from CO_2_ fertilization effects and extended growing seasons. Maps represent ensemble model outputs from the years 2020 to 2090. References: [210,211,212,213,214,215,216,217,218,219,220,221,222].

In response, processors may increase alkali concentration, extraction temperature, or residence time, all of which amplify environmental burdens and risk undesirable modification of protein structure [209]. Alternatively, blending raw materials across global regions may be used to stabilize functionality (Figure 4), but this strategy increases transportation impacts and complicates traceability.

### 5.3. Climate Impacts on Functional Performance and Texturization

Beyond extraction yield, climate-driven changes in protein structure have significant implications for ingredient functionality. Proteins synthesized under stress conditions often display altered secondary and tertiary structures, influencing hydration kinetics, interfacial adsorption, and aggregation behavior [223]. Even modest shifts in amino acid composition or protein folding can translate into substantial differences in solubility, emulsification, and gelation performance [125].

In beverage and dairy-alternative applications, increased aggregation propensity may exacerbate sedimentation and phase instability, increasing reliance on hydrocolloids or mechanical homogenization [160,162,171]. In meat analogs, variability in protein viscoelastic behavior can affect melt flow during extrusion or shear-cell processing, leading to inconsistent fiber formation, anisotropy, and texture [164,224].

Climate-driven variability therefore increases process sensitivity and reduces tolerance windows, making PBP manufacturing more complex and less predictable [225,226]. Maintaining consistent product quality under these conditions often requires increased formulation buffers or processing intensity, with attendant sustainability trade-offs.

### 5.4. Interaction Between Climate Change and Food Safety Risks

Climate change amplifies food safety risks throughout PBP value chains. Elevated temperatures and altered precipitation patterns increase fungal infection pressure in crops, raising the prevalence and geographic distribution of mycotoxin-producing species. Drought stress weakens plant defenses, while insect damage creates entry points for fungal colonization, further increasing contamination risk [227,228].

Post-harvest storage is increasingly challenged by warmer and more humid conditions, particularly in regions lacking controlled storage infrastructure. Mycotoxins are of particular concern because many are chemically stable and resistant to thermal processing [229]. Protein concentration and isolation processes may therefore retain or concentrate mycotoxins within protein-rich fractions if raw materials are inadequately screened.

Climate-driven shifts in soil and crop microbiomes may also increase the prevalence of spore-forming bacteria, complicating microbial control in plant-based foods [11,230,231]. Together, these factors necessitate climate-adaptive food safety strategies embedded within sustainability frameworks rather than treated as independent concerns.

### 5.5. Consequences for Sustainability Assessment and System Resilience

Climate-induced changes in protein composition, processing performance, and safety risk undermine static sustainability assessments that assume stable crop composition and processing efficiency. LCAs that do not account for declining protein concentration, increased processing energy per unit protein, or elevated reject rates due to safety concerns may significantly underestimate future environmental burdens.

Resilience to climate variability therefore becomes a critical dimension of sustainability for plant-based protein systems. Strategies that improve resilience—such as diversification of protein sources, flexible processing technologies, and climate-resilient crop breeding—may deliver long-term sustainability benefits even if their short-term environmental footprints appear higher.

Recognizing climate change as a value-chain-wide driver rather than an upstream agricultural issue is essential for realistic assessment and design of sustainable plant-based protein foods.

## 6. Nutrition and Health Implications of Plant-Based Proteins

PBP foods are increasingly positioned as nutritionally equivalent or superior alternatives to animal-derived proteins, particularly in the context of environmental sustainability and chronic disease risk reduction. However, nutritional adequacy depends not only on total protein intake but on protein quality, digestibility, bioavailability of essential nutrients, and interactions with the broader food matrix [57,232,233,234]. These factors are further modulated by processing intensity and climate-driven changes in crop composition, complicating one-to-one substitution of animal proteins with plant-based alternatives [235,236,237,238].

### 6.1. Protein Quality Beyond Crude Protein Content

Crude protein concentration is an incomplete indicator of nutritional adequacy (Table 1). Protein quality depends on the presence and relative proportions of essential amino acids, their digestibility, and their availability for human metabolism [239,240,241]. In contrast to animal proteins, which often provide essential amino acids in proportions closely aligned with human requirements, many plant proteins are limited in one or more essential amino acids [242].

Cereal proteins are commonly deficient in lysine [243], while legume proteins are often limited in sulfur-containing amino acids such as methionine and cysteine [102]. Oilseed proteins may present additional limitations depending on cultivar and processing history. These patterns necessitate dietary complementarity—either through consumption of multiple plant protein sources or through formulation strategies that blend proteins at the ingredient level.

Climate-driven changes exacerbate these compositional constraints. Elevated atmospheric CO_2_ and temperature stress have been associated with reductions in essential amino acids, particularly sulfur-containing amino acids, even when total protein content appears unchanged [244]. These amino acids play critical roles in protein synthesis, antioxidant defense, methylation reactions, and maintenance of muscle mass [245]. Declines in their availability therefore have implications for vulnerable populations such as children, older adults, and pregnant individuals.

From a sustainability perspective, deterioration of protein quality undermines assumptions used in dietary modeling and life-cycle assessment, where protein quantity is often treated as interchangeable across sources. If higher quantities of plant protein are required to achieve equivalent nutritional outcomes, environmental advantages may be partially eroded by increased production and processing demands.

### 6.2. Digestibility Constraints and Bioavailability

Digestibility is a central determinant of protein nutritional value and varies widely among plant protein sources (Table 1). Plant proteins are typically embedded within complex matrices containing dietary fiber, cell wall structures, and antinutritional compounds such as phytates, tannins, lectins, and protease inhibitors. These components limit enzymatic access to protein substrates and reduce both protein and mineral absorption [246,247,248,249].

Processing can substantially modify digestibility. Thermal treatment unfolds protein structures and inactivates protease inhibitors, while extrusion combines heat, shear, and pressure to disrupt cellular matrices and improve enzyme accessibility [250,251]. Fermentation can degrade phytates and polyphenols, enhance mineral bioavailability, and partially hydrolyze proteins, improving digestibility [252]. Enzymatic treatments directly cleave peptide bonds, increasing amino acid availability but potentially altering functional properties [253].

However, improvements in digestibility often come with trade-offs. Intensive thermal or chemical processing can induce Maillard reactions, oxidize amino acids, or promote irreversible aggregation that reduces bioaccessibility [254]. Enzymatic hydrolysis may increase bitterness or compromise texture. Consequently, optimizing digestibility requires careful balancing of nutritional gains against sensory and functional performance.

Assessment of protein digestibility remains methodologically challenging. In vitro digestion models provide useful screening tools, but often fail to replicate human gastrointestinal dynamics [255]. Animal models capture physiological complexity but raise ethical and translational concerns. Human studies offer the highest relevance but are expensive, time-consuming, and sensitive to interindividual variability. Digestibility is further influenced by food matrix structure, particle size, lipid content, and co-ingested nutrients, underscoring the difficulty of assigning fixed digestibility values to protein ingredients [256].

### 6.3. Micronutrient Interactions and Antinutritional Factors

Beyond protein, PBP foods must deliver adequate levels of essential micronutrients to support nutritional equivalence. Iron, zinc, calcium, iodine, and vitamin B_12_ are of particular concern in plant-based diets [257]. Many plant proteins are intrinsically low in these nutrients or contain them in forms with limited bioavailability.

Phytates present in legumes and cereals strongly chelate multivalent minerals, reducing absorption [258]. Polyphenols and dietary fiber further inhibit mineral uptake under certain conditions [259]. Climate change compounds these challenges by reducing mineral concentrations in crops through carbon dilution and altered soil nutrient dynamics [186].

Processing can mitigate some micronutrient limitations. Fermentation reduces phytate content and enhances mineral bioavailability, while fortification strategies can compensate for intrinsic deficiencies [260]. However, fortification introduces additional sustainability considerations related to sourcing, formulation stability, and regulatory compliance [261].

Importantly, micronutrient deficiencies may arise not from inadequate intake but from impaired absorption [262]. Sustainability assessments that focus solely on nutrient content without accounting for bioavailability risk overestimate the nutritional performance of PBP foods [259].

### 6.4. Health Outcomes and Epidemiological Evidence

Epidemiological studies consistently associate plant-rich dietary patterns with reduced risk of cardiovascular disease, improved lipid profiles, and lower all-cause mortality [263]. Diets emphasizing legumes, whole grains, nuts, and soy products are linked to lower LDL cholesterol and improved glycemic control [264]. However, these associations reflect whole-diet effects rather than isolated contributions of plant proteins. Plant-based diets typically differ from omnivorous diets in multiple dimensions, including fiber intake, fatty acid composition, energy density, and consumption of ultra-processed foods [265]. Disentangling the specific impact of plant protein per se from these co-varying factors remains challenging.

Randomized controlled trials comparing plant and animal protein isolates generally reveal modest or neutral differences in cardiometabolic markers when protein dose and energy intake are matched [266,267]. In some contexts, animal proteins show slightly greater stimulation of muscle protein synthesis, particularly in older adults, due to higher leucine content and faster digestibility [268]. These differences may be mitigated through higher intake levels, protein blending, or targeted supplementation.

Claims regarding the inherent health superiority of PBP must therefore be interpreted cautiously and contextualized within broader dietary patterns.

### 6.5. Ultra-Processing, Functionality, and Nutrition Trade-Offs

A recurring critique of PBP foods is their classification as ultra-processed. While processing intensity varies widely across products, many PBP foods rely on significant processing to overcome the functional and nutritional limitations of raw materials. From a nutritional standpoint, processing can be both beneficial and detrimental.

On one hand, processing improves digestibility, inactivates antinutritional factors, and enhances safety and shelf life [269]. On the other hand, excessive refining may remove beneficial components, increase sodium content, or reduce nutrient density [270]. The relationship between processing, nutrition, and health is therefore not linear. Simplistic narratives equating minimal processing with superior nutrition fail to account for the functional reality of plant proteins. In many cases, adequate nutrition depends on appropriate processing, particularly when replacing nutrient-dense animal foods.

### 6.6. Climate Change and Long-Term Nutritional Resilience

Climate-induced changes in crop composition threaten the long-term nutritional adequacy of PBP systems. Reductions in protein quality, micronutrient density, and digestibility may silently erode the nutritional foundation of plant-based diets, particularly in regions already facing food insecurity [271].

Nutritional resilience must therefore be considered alongside environmental sustainability. Crop breeding strategies that prioritize protein quality and micronutrient retention, processing approaches that preserve bioavailability, and dietary guidelines that emphasize protein complementarity will be essential for maintaining health outcomes under changing climatic conditions [271].

From a systems perspective, nutrition is both an outcome and a constraint: PBP foods that fail to deliver adequate nutrition cannot be considered sustainable, regardless of their environmental footprint.

## 7. Food Safety and Sustainability Interactions in Plant-Based Protein Systems

Plant-based protein foods are frequently perceived by consumers as inherently safer and healthier than animal-derived products, a perception reinforced by concerns surrounding zoonotic disease, antimicrobial resistance, and intensive animal production [272]. However, accumulating evidence demonstrates that PBP systems face distinct but equally complex microbial, chemical, and allergenic risks, many of which are intimately linked to sustainability considerations [273]. These risks are dynamic rather than static, evolving in response to crop selection, climate variability, processing intensity, formulation strategies, and the globalization of food supply chains (Figure 1). Consequently, food safety must be regarded as a foundational dimension of sustainability, rather than as a downstream constraint addressed independently of environmental and nutritional objectives.

### 7.1. Microbial Hazards Across the Plant-Based Protein Value Chain

Protein-rich plant crops are exposed to diverse and highly variable microbial communities during cultivation, harvest, storage, and transportation. Unlike animal-derived foods, which often pass through centralized and highly regulated slaughter and processing facilities, plant-based protein raw materials are typically sourced from decentralized agricultural systems spanning broad geographic regions. This decentralization increases variability in microbial load and composition, particularly when crops are aggregated across farms, regions, and climates.

Climate-driven increases in temperature extremes, prolonged drought, and erratic precipitation patterns substantially alter soil and crop microbiomes [274,275,276]. Heat and moisture stress favor the proliferation of stress-tolerant and opportunistic microorganisms, while suppressing beneficial microbial communities that contribute to plant health [277]. These changes increase the prevalence of spoilage organisms and opportunistic pathogens on raw materials entering the plant-based protein supply chain.

Many commercially relevant PBP foods, including dairy alternatives, protein beverages, meat analogs, and ready-to-eat products, occupy low-acid, moderate-moisture matrices that can support microbial survival and growth if processing and formulation hurdles are insufficient [272,278]. Controlled studies have demonstrated that products such as plant-based milks, yogurts, nut butters, protein gels, and hybrid foods can support the growth of pathogens, including *Listeria monocytogenes*, *Salmonella* spp., and *Escherichia coli*, under temperature-abuse conditions [11,230,231,279,280,281].

Particularly concerning are spore-forming bacteria, including *Bacillus* and *Paenibacillus* species [11]. These organisms are ubiquitous in soil, highly resistant to thermal treatments, and capable of surviving pasteurization, extrusion, or other heat-based processes. Spores may germinate during storage or distribution, especially when competing microflora are suppressed by processing or preservatives. Climate change is expected to exacerbate the prevalence and resilience of spore-forming bacteria, increasing the burden on food safety management systems.

### 7.2. Clean-Label Trends and Unintended Safety Trade-Offs

Sustainability-driven consumer preferences increasingly emphasize “clean-label” products with fewer ingredients and minimal processing. While reduced additive use may align with consumer perception of naturalness and environmental responsibility, such trends can inadvertently compromise food safety. Ingredients such as salt, organic acids, sugars, and stabilizers often serve as important safety hurdles, reducing water activity, lowering pH, or stabilizing product structure in ways that can inhibit microbial growth [282].

Eliminating these components without compensatory design measures can create conditions favorable to pathogen survival or growth, particularly in plant-based foods that lack inherent antimicrobial barriers [272]. Thus, reducing formulation complexity does not necessarily improve sustainability if it increases spoilage, food waste, or foodborne illness risk [278]. In many cases, appropriate processing and formulation complexity enhance overall sustainability by extending shelf life, improving safety, and reducing downstream waste.

### 7.3. Mycotoxins and Climate-Driven Chemical Hazards

Chemical hazards, particularly mycotoxins, represent one of the most significant food safety challenges for plant-based protein systems [283,284,285]. Legumes, cereals, and oilseeds are susceptible to colonization by toxin-producing fungi both pre- and post-harvest [286]. Climate change is expanding the geographic range, incidence, and severity of fungal contamination through several mechanisms, including heat stress, drought-induced plant weakening, and increased insect damage that facilitates fungal ingress [287].

Mycotoxin occurrence is strongly influenced by environmental stress [288]. Drought and heat stress weaken plant defenses, increasing susceptibility to fungal infection, while extreme rainfall events can exacerbate post-harvest contamination during drying and storage. As these conditions become more frequent, mycotoxin prevalence is expected to increase, particularly for crops used extensively in plant-based protein production.

Protein concentration and isolation processes do not reliably eliminate mycotoxins. Many mycotoxins are chemically stable and resistant to thermal processing, extrusion, and drying [229]. In some cases, fractionation may actually concentrate mycotoxins if contaminants partition preferentially into protein-rich streams or if co-products are inadequately monitored [289]. As consumption of PBP concentrates and isolates increases, cumulative dietary exposure to mycotoxins may rise unless comprehensive raw material screening, segregation, and processing controls are implemented.

From a sustainability perspective, failure to control mycotoxin risk has cascading consequences. Product recalls, regulatory non-compliance, increased food waste, and loss of consumer trust undermine environmental, economic, and social sustainability goals simultaneously.

### 7.4. Processing Intensity as a Safety–Sustainability Lever

Processing technologies play a dual role in plant-based protein systems. They are essential for achieving desired functionality and nutritional performance, while simultaneously serving as primary tools for hazard mitigation. Thermal processing, fermentation, enzymatic treatment, and extrusion reduce microbial load, inactivate antinutritional factors, and improve protein digestibility. However, these benefits must be balanced against the environmental impacts associated with energy and water consumption.

The assumption that less processing equates to greater sustainability is therefore flawed. In many instances, adequately designed processing enhances system-level sustainability by reducing spoilage, extending shelf life, and preventing foodborne illness [290]. Conversely, under-processing may reduce immediate energy use while increasing long-term waste, recall risk, and human health burden [291].

The sustainability profile of PBP foods should thus be evaluated based on net system performance, accounting for safety, waste reduction, and nutritional adequacy rather than processing intensity alone.

### 7.5. Allergenicity as a Sustainability Concern

Allergenicity constitutes another critical interface between food safety and sustainability in PBP systems. Soy, wheat gluten, and several legumes are regulated allergens in many jurisdictions. Climate stress, crop breeding, and genetic modification may alter expression levels and profiles of allergenic proteins, while processing can denature, expose, or aggregate allergenic epitopes in unpredictable ways [292].

As PBP portfolios diversify and global consumption increases, allergen management becomes increasingly complex. Cross-contamination risks rise with shared processing infrastructure, and accurate labeling becomes essential for consumer safety [293]. Failure to integrate allergen management into sustainability assessments risks undermining social equity by limiting safe food access for allergic populations [294].

In a broader context, sustainability frameworks that exclude allergenicity overlook an important dimension of consumer health, regulatory compliance, and social inclusion.

## 8. System-Level Synthesis and Future Directions

### 8.1. Sustainability as an Emergent System Property

**Figure 5:**
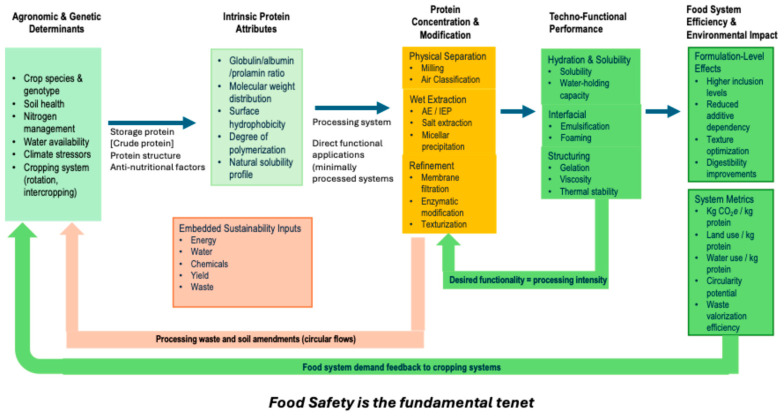
Integrated framework linking plant-based protein functional properties with sustainability determinants. References: Legumes and Beans: [13,14,15,16,17,18,19,20,21,22,23]; Cereals: [24,25,26,27]; General: [28,29,30,31,32].

A central conclusion of this review is that sustainability in PBP foods cannot be attributed to individual crops, ingredients, or processing technologies in isolation. Instead, sustainability emerges from interactions among biological, technological, nutritional, safety, and socio-economic factors operating across interconnected scales (Figure 5). Crop genetics determine protein composition and stress tolerance; agronomic practices shape environmental inputs; processing technologies influence resource use, functionality, and safety; formulation strategies affect nutrition and consumer acceptance; and climate variability perturbs all components simultaneously.

This systems perspective challenges reductionist narratives that frame sustainability primarily in terms of GHG*e* per kilogram of product. While such metrics are informative, they obscure critical trade-offs. A PBP ingredient with a low carbon footprint but poor nutritional adequacy or elevated food safety risk cannot be considered sustainable in a holistic sense. Conversely, processing interventions that increase energy use may improve digestibility, reduce spoilage, and enhance safety, yielding net positive sustainability outcomes when evaluated across the full life cycle.

Understanding sustainability as an emergent property has practical implications. It shifts focus away from identifying “best” protein sources and toward designing resilient protein systems capable of delivering safe, nutritious foods under dynamic environmental and market conditions.

### 8.2. Climate Change as a Force Multiplier Across Protein Systems

Climate change acts as a force multiplier across plant-based protein value chains. Elevated atmospheric CO_2_, increased temperature variability, altered precipitation regimes, and extreme events interact to reshape crop physiology, protein composition, and contaminant risk. These upstream changes cascade through processing, formulation, and distribution, amplifying variability and uncertainty at each downstream step.

Carbon dilution reduces protein and micronutrient concentrations precisely as global demand for PBP increases [8,9,10]. Heat and drought stress alter amino acid profiles and storage protein ratios, affecting both nutritional quality and functional performance [295,296]. At the same time, climate stress elevates fungal infestation, mycotoxin prevalence, and microbial hazards, increasing food safety burdens on processors and regulators [297,298].

These converging pressures undermine assumptions of compositional stability that underpin many current sustainability assessments, nutritional models, and processing designs. Addressing climate impacts therefore requires anticipatory adaptation rather than reactive adjustment [299,300]. Climate resilience must be incorporated explicitly into sustainability frameworks for PBP systems.

### 8.3. Implications for Sustainability Assessment and Life-Cycle Analysis

Most existing LCAs of PBP foods assume stable protein concentration, consistent processing yields, and uniform nutritional equivalence over time. The evidence reviewed here suggests that these assumptions are increasingly untenable under climate change. Declining protein concentration increases energy and water use per unit of edible protein, while compositional variability necessitates higher processing intensity or raw material blending [301].

Additionally, LCAs often omit food safety losses, nutrition-adjusted functional units, and climate-driven reject rates [119]. Incorporating metrics such as protein quality-adjusted yield, bioavailable nutrient density, and losses due to contamination or spoilage would improve the realism and policy relevance of sustainability assessments.

Future sustainability modeling must therefore move beyond static inventories and adopt dynamic, systems-oriented approaches that account for climate variability, nutritional adequacy, and safety outcomes.

### 8.4. Research Priorities at the Crop–Processing–Nutrition Interface

Several cross-cutting research priorities emerge from this synthesis. First, climate-resilient crop breeding must extend beyond yield stability to include protein quality, amino acid composition, and micronutrient retention under elevated CO_2_ and combined heat–drought stress. Integrating nutritional targets into breeding programs is essential for preserving the long-term viability of plant-based diets.

Second, adaptive and resource-efficient processing technologies are needed to accommodate increased variability in raw material composition. Processing systems that are robust to fluctuations in protein fractionation, solubility, and aggregation behavior will be critical for maintaining product consistency without excessive energy or water use.

Third, standardization of functional and nutritional metrics is urgently needed. Harmonized methods for measuring solubility, digestibility, amino acid bioavailability, and functional performance would improve comparability across studies and accelerate translation of research into industrial practice.

Lastly, integration of food safety into sustainability research must become routine. Mycotoxins, spore-forming bacteria, and allergen risks are integral to system performance and should be incorporated explicitly into sustainability assessments rather than treated as separate regulatory issues.

### 8.5. Implications for Food Industry Strategy

For the industry, the findings of this review highlight the importance of system-level design rather than ingredient-centric optimization. Relying on a narrow set of protein crops or rigid processing parameters increases vulnerability to climate variability and supply disruptions. Diversification of protein sources, flexible processing configurations, and adaptive formulation strategies can enhance resilience even if they introduce modest short-term environmental costs.

Industry responses to clean-label and minimal-processing trends should also be tempered by safety and nutritional considerations. Processing decisions framed solely around consumer perception risk increasing spoilage, waste, and health hazards. Long-term sustainability depends on balancing environmental goals with robust safety margins and nutritional performance.

### 8.6. Policy and Governance Considerations

Policy initiatives promoting plant-based diets often emphasize environmental benefits without sufficient consideration of nutrition and food safety. Dietary guidelines, sustainability labeling schemes, and public procurement standards should reflect nutrition-adjusted and safety-aware sustainability metrics, rather than emissions alone.

Investments in infrastructure for raw material monitoring, climate-adaptive storage, and food safety surveillance will be particularly important in regions where PBP consumption is rising faster than regulatory capacity. Failure to address these needs risks undermining public confidence in plant-based foods and exacerbating inequities in food access.

### 8.7. Concluding Perspective

Plant-based protein foods will play an essential role in future food systems, particularly in efforts to reduce the environmental impacts of animal agriculture. However, their sustainability cannot be assumed. Climate change is already reshaping the biological foundations of plant-derived proteins, with cascading effects on processing performance, food safety, and nutritional quality.

Recognizing sustainability as a dynamic, emergent property of interacting systems enables more realistic evaluation and more effective intervention. By integrating crop science, food processing, functional design, safety management, nutrition, and climate resilience, stakeholders can design plant-based protein systems that are environmentally sound, nutritionally adequate, socially equitable, and robust under uncertain future conditions.

## Figures and Tables

**Table 1:** Nutritional properties and digestibility of PBP ^1^.

Protein Source	Crude Protein (%)	PDCAAS ^2^	DIAAS ^2^	Digestibility (%)	Limiting Amino Acids (In Relative Order)	Amino Acids as Good Source	Anti-Nutritional Factors ^3^
Soybean	36–56	0.9–1.0	80–90	89–95	Met, Cys, Lys	Lys (vs. cereals)	Phytic acid, lectins, saponins, oligosaccharides, and trypsin/chymotrypsin inhibitors
Wheat	12–15	0.4–0.45	~20	90–93	Lys	Met, Cys, Leu	Phytic acid, enzyme inhibitors, lectins, tannins
Yellow pea	19–25	0.6–0.7	~60–70	85–100	Trp, Met, Cys	Lys	Phytates, tannins, lectins, ⍺-galactosides, trypsin/chymotrypsin inhibitors
Rice	2–3	0.6	37–42	85–100	Lys	Met, Cys	Phytic acid, phenolic compounds, enzyme inhibitors
Chickpea	8.8–9.5	0.8–0.85	~40–60	75–89	Met, Cys, Trp	Lys, Leu, Ile	Phytic acid, saponins, ⍺-galactosides, tannins, trypsin inhibitors
Faba bean	25–30	0.6–0.65	~30–60	80–85	Met, Cys, Trp	Lys, Leu, Ile, Thr	Vicine/convicine, phytic acid, lectins, tannins, trypsin inhibitors
Yellow lentil	22–27	0.5–0.6	~58	66–86	Trp, Lys	Leu, Ile, Val, Lys	Phytic acid, tannins, trypsin inhibitors, lectins, ⍺-galactosides
Corn	3.3–3.5	0.4–0.5	<50–60	62–90	Lys, Trp	Leu, Ile, Val, Met, Cys	Phytic acid, tannins

^1^ Values are for proteins extracted from individual plant species; values with ranges reflect the level of processing after extraction. Generally, more processing increases DIAAS. ^2^ Abbreviations: PDCAAS: Protein Digestibility Corrected Amino Acid Score; DIAAS: Digestible Indispensable Amino Acid Score. ^3^ Antinutrients cause plant-based foods to be poorly digested through enzyme inhibition or by sequestering the minerals Fe, Zn, Ca, and Mg. References: Legumes and Beans: [13,14,15,16,17,18,19,20,21,22,23]; Cereals: [24,25,26,27]; General: [28,29,30,31,32].

**Table 3:** Environmental Consequences of Protein-rich Crops Commonly Used as Food Ingredients for Feeding Animals and Humans ^1^.

Crop	Land Use Harvested (M ha)	Total Water Footprint (m^3^/T Grain)	GHG*e* (kg CO_2_ *eq*/kg Grain)	Energy Use (GJ/T)	Atmospheric N_2_O (Kg N/ha/yr)	Eutrophication Index ^2^ M: kg N-*eq*/ha F: kg P-*eq*/ha
Soybeans	34.85	2249	0.3–0.4	1.1–2.2	0.7–1.85	M: 0.38–0.80 F: 0.16–0.21
Wheat	15.6	500–4000	0.7	3.1–4.9	0.3–1.5	M: 0.06–0.99 F: 0.135–3.04
Total Peas	0.44	1979	0.27	7.02–9.7	0.4–1.7	M: 5.0–12.0 F: 0.3–1.0
Rice	1.15	1163	0.84–1.79	10–12	0.65–9.1	M: 0.06–0.41 F: 1.24–1.33
Chickpeas	0.212	12,227	1.46	3–7	0.13–0.17	M: 1.03 × 10^−11^ F: 0.20–1.20
Faba Bean	0.016	638	0.23–0.71	4.25–5.6	0.3–1.5	M: <15 F: 0.1–1.5
Total Lentils	0.384	1250	0.72–0.9	1.3–1.6	0.7–1.33	M: 0.5–5.0 F: 0.1–2.0
Corn	34.5	489	0.12–0.44	1.5–2.5	2.3–7.8	M: 10–60 F: 0.5–5

^1^ Globally, values vary in most categories because of soil and climatic conditions, methods of farming, and agronomic characteristics of seed stock. ^2^ Abbreviations: M: marine water; F: freshwater. References: Soybean: [41,42,43,48,92,93,94]; Wheat: [43,53,92,95,96]; Total peas: [43,58,97]; Rice: [43,92,98,99,100,101,102,103]; Chickpeas: [43,59,68,104,105]; Faba bean: [43,59,71,106,107]; Lentil: [43,108,109,110]; Corn: [43,81,92,111,112,113,114,115,116].

## Data Availability

No new data were created or analyzed in this study.
